# Deregulated Serotonin Pathway in Women with Morbid Obesity and NAFLD

**DOI:** 10.3390/life10100245

**Published:** 2020-10-16

**Authors:** Jessica Binetti, Laia Bertran, David Riesco, Carmen Aguilar, Salomé Martínez, Fàtima Sabench, Jose Antonio Porras, Javier Camaron, Daniel Del Castillo, Cristóbal Richart, Teresa Auguet

**Affiliations:** 1 Grup de Recerca GEMMAIR (AGAUR)—Medicina Aplicada, Departament de Medicina i Cirurgia, Institut d’Investigació Sanitària Pere Virgili (IISPV), Universitat Rovira i Virgili (URV), 43007 Tarragona, Spain; jessica.binetti@gmail.com (J.B.); laia.bertran@urv.cat (L.B.); caguilar.hj23.ics@gencat.cat (C.A.); aporras.hj23.ics@gencat.cat (J.A.P.); cristobalmanuel.richart@urv.cat (C.R.); 2 Servei Medicina Interna, Hospital Universitari de Tarragona Joan XXIII, 43007 Tarragona, Spain; driesco.hj23.ics@gencat.cat (D.R.); javiercamaron93@gmail.com (J.C.); 3 Servei Anatomia Patològica, Hospital Universitari de Tarragona Joan XXIII, 43007 Tarragona, Spain; mgonzalez.hj23.ics@gencat.cat; 4 Servei de Cirurgia, Hospital Universitari Sant Joan de Reus, Departament de Medicina i Cirurgia, Institut d’Investigació Sanitària Pere Virgili (IISPV), Universitat Rovira i Virgili (URV), 43204 Reus, Spain; fatima.sabench@urv.cat (F.S.); danieldel.castillo@urv.cat (D.D.C.)

**Keywords:** serotonin, non-alcoholic fatty liver disease, morbid obesity, bariatric surgery, metabolic syndrome

## Abstract

Non-alcoholic fatty liver disease (NAFLD) extends from simple steatosis (SS) to non-alcoholic steatohepatitis (NASH). Peripheral serotonin (5-HT) has become as an important regulator of different metabolic pathways. 5-HT has been related to obesity and lipid accumulation in the liver. The objective of this study was to assess the relationship between the 5-HT signaling pathway and the degree of NAFLD, as well as to investigate whether peripheral 5-HT levels are related to the hepatic and jejunal mRNA abundance of serotonin receptors (HTR) in a cohort of women with morbid obesity (MO) and NAFLD. ELISA was used to quantify the serum 5-HT from normal-weight subjects (n = 26) and patients with MO (n = 58). We used RTq-PCR analysis to evaluate the relative expression of HTR in women with MO with normal liver (n = 22), SS (n = 21), and NASH (n = 15). The 5-HT was diminished in women with MO under a hypocaloric diet, regardless of the presence of NAFLD. Additionally, we report a negative correlation of 5-HT levels with metabolic syndrome criteria, suggesting that serotonin may have a protective role in obesity. Additionally, the hepatic expression of HTR2A and HTR2B were decreased in women with MO and NAFLD, but no significant differences in the HTR jejunal expression according to the presence of NAFLD were found.

## 1. Introduction

Non-alcoholic fatty liver disease (NAFLD) is a current health problem that affects people worldwide, and is a main cause of liver transplantation and hepatocellular carcinoma [1]. In recent years, it has been reported that the clinical relevance of NAFLD is not only focused on liver-related morbidity and mortality. NAFLD has become a multisystemic disease, affecting extra hepatic tissues, and is linked to metabolic complications such as type 2 diabetes mellitus (T2DM) and cardiovascular disease independent of other risk factors. Moreover, recent studies have suggested a possible relationship between NAFLD and depressive disorders [2]. In this sense, treatment for obesity, T2DM, or even anti-depressant drugs in selected NAFLD cases should be useful in treating NAFLD [3]; nevertheless, specific pharmacological treatments for NAFLD have not yet been approved [4,5,6]. Therefore, improving our knowledge of molecular pathways involved in the progression of NAFLD is needed to identify future therapeutic targets.

The NAFLD progression mechanism is usually explained with the “multiple hit” theory of NAFLD pathogenesis, which states that lipid accumulation triggers liver steatosis, bringing about other processes such as adipokine release, inflammation, lipotoxicity, glucose, and lipid homeostasis dysregulation, which end up causing non-alcoholic steatohepatitis (NASH) and cirrhosis. Systemic inflammation is linked to progression to NASH, but it is also related to other pathological processes, such as innate immunity alterations, oxidative stress, mitochondrial dysfunction, toll-like receptors activation, and gut dysbiosis [7]. In this sense, the gut–liver axis plays a key role in the progression of NAFLD. Changes in the microbial composition generated by a high-fat diet (HFD) or high-carbohydrate diet (HCD) give rise to a release of metabolites derived from the microbiota that have a negative impact on liver metabolism, promoting the development of metabolic diseases such as NAFLD [8]. On the other hand, the release into the bloodstream of histamine synthesized by intestinal mast cells [9], or enterochromaffin (EC) cell-derived serotonin [10] also promote liver metabolic dysregulation, leading to steatosis and liver inflammation.

Serotonin (5-hydroxytryptamine, 5-HT) is a monoamine neurotransmitter that regulates central and peripheral functions. The central and peripheral 5-HT mechanisms of action function separately, since 5-HT cannot cross the blood–brain barrier [11]. Therefore, 5-HT plays a role not only as a neuronal neurotransmitter controlling mood, sleep, and anxiety but also as a relevant regulator molecule in the gastrointestinal tract and other organs [12].

Most of the peripheral 5-HT is produced in the gut. 5-HT biosynthesis is a rate-limited process carried out by tryptophan hydroxylase isoform (TPH1) in intestinal EC cells, which respond to mechanical stimuli, glycemic status, and microbial digestion products [12]. The physiological actions of peripheral 5-HT occur through the activation of more than 14 serotonin receptor subtypes (HTRx) of seven families [13] located on different cell types such as enterocytes [14], enteric neurons [15], or immune cells [16]. HTR receptors are G-protein-coupled receptors except for serotonin receptor 3 (HTR3), which is a ligand-gated caption channel [17,18].

Once released into the bloodstream, most 5-HT is sequestered into platelets and distributed to different parts of the body [19]. The rest of the 5-HT goes through the systemic circulation and arrives at peripheral tissues in free form. The free 5-HT levels in systemic circulation are very low because most of them are metabolized in the liver and lungs [11]. These gut-derived 5-HT modulate different actions, such as enteric motor and secretory reflexes [13], platelet aggregation [20], and immune responses [16], among others. The dysregulation of 5-HT levels has been involved in the pathogenesis of diseases such as irritable bowel syndrome [21], cardiovascular disease, and osteoporosis [22].

**Figure 1:**
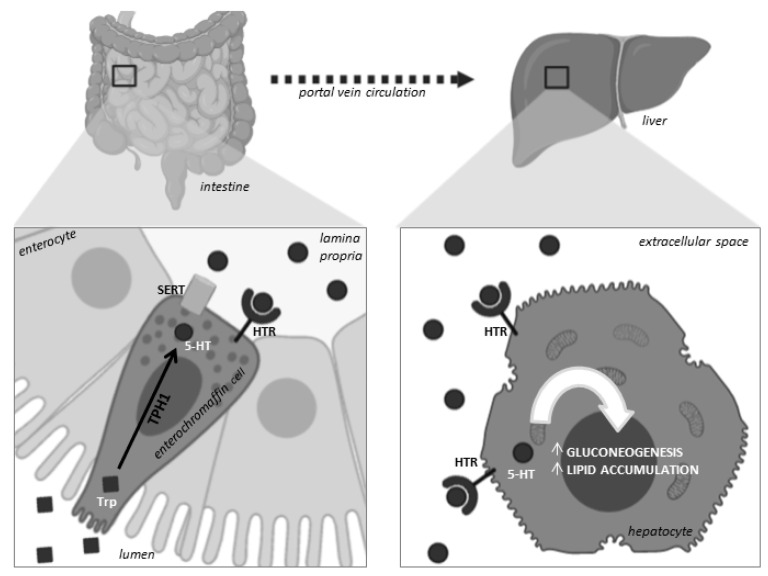
Gut-derived serotonin mechanism of action of hepatocytes. TPH1 enzyme synthesizes 5-HT in enterochromaffin cells from tryptophan captured from the intestinal mucosa. This 5-HT is released and travels through the portal vein circulation to the liver, where it is captured by hepatocytes HTR and promotes lipid accumulation and gluconeogenesis. These processes result in the progression of hepatic steatosis. 5-HT, serotonin; SERT, serotonin transporter; TPH1, tryptophan hydroxylase; HTR, 5-HT receptor; Trp, tryptophan.

Peripheral serotonin has become an important regulator of energy metabolism, carrying out diverse physiopathological roles in multiple metabolic tissues [23,24,25]. 5-HT has been known to promote gluconeogenesis and lipid accumulation in hepatocytes in vitro, as shown in Figure 1 [26]. In response to fasting, the 5-HT levels increase considerably due to the overexpression of TPH1. High levels of 5-HT promote lipolysis in adipocytes that generate substrates for hepatic gluconeogenesis [25]. Additionally, high levels of 5-HT modulate glucose metabolism, enhancing pancreatic insulin secretion, which gives rise to insulin resistance that promotes hepatic steatosis [27,28]. Recently, an in vitro and in vivo study has demonstrated that 5-HT regulates hepatocarcinoma steatosis and may enhance hepatic carcinogenesis [29]. Furthermore, the inhibition of hepatic serotonin receptor 2A (HTR2A) signaling in vivo by blocking the synthesis of 5-HT improves liver steatosis [28], as well as hyperglycemia and dyslipidemia [30]. 5-HT signaling has recently been studied in human intestinal mucosal biopsies, and it has been reported that HTR3 and HTR4 may act as gastrointestinal sensory and motor function receptors [31,32], which may impact liver metabolism through the gut–liver axis.

Despite all this, regarding obesity there are controversies [33,34]. On one hand, peripheral 5-HT inhibits adaptive thermogenesis process, also called browning, by inactivating the hormone-sensitive lipase and reducing the expression of uncoupling protein 1 [23,24]. In contrast, high levels of 5-HT have been reported in humans with obesity [34] and in rodents with a HFD [35]. This may be due to the fact that, in obesity conditions, there is a higher density of EC cells and an increased expression of TPH1 [34]; however, the role of 5-HT in obesity still remains unclear.

Therefore, the first objective of the present work is to elucidate the role of 5-HT in obesity. Moreover, as the relationship between the serum 5-HT and its liver and gut receptors is poorly studied in humans with NAFLD, the second aim was to assess the relationship between the 5-HT signaling pathway and the degrees of NAFLD. We further aim to investigate whether the peripheral 5-HT levels are related to the hepatic and jejunal mRNA abundance of serotonin receptors (HTR) in a cohort of women with morbid obesity (MO) and NAFLD.

## 2. Materials and Methods

### 2.1. Study Subjects

The study was approved by the institutional review board, and all participants gave written informed consent (23c/2015).

The study population consisted of 84 Caucasian women: 26 NW controls (BMI < 25 kg/m^2^) without caloric restrictions and 58 patients with MO (BMI > 40 kg/m^2^) who have undergone a very low caloric diet (VLCD) from 3 months before bariatric surgery [36]. VLCD is based on administering up to 800 kcal/day for a period prior to bariatric surgery in order to decrease the risk of complications and increase the chances of long-term success. Obese patients under a VLCD lost an average weight of 7.65 ± 2.66 kg.

On the other hand, as men and women differ substantially in regard to body composition, energy imbalance, hormones, and lipid and glucose metabolism [37], and also sexual dimorphism in NAFLD seems to exist [38], we included only women in our study in order to avoid the interference of gender.

Liver and jejunal biopsies from patients with MO were obtained during planned laparoscopic bariatric surgery. All the liver biopsies were indicated for clinical diagnosis. The exclusion criteria were as follows: (1) subjects who had alcohol consumption higher than 10 g/day; (2) patients who had acute or chronic hepatic, inflammatory, infectious, or neoplastic diseases; (3) women who were menopausal or undergoing contraceptive treatment; (4) diabetic women receiving pioglitazone, glucagon-like peptide-1 (GLP-1) receptor agonists, dipeptidyl peptidase-4 (DPP-4) inhibitors, or insulin; (5) patients treated with antibiotics in the previous 4 weeks or receiving cholestyramine or ursodeoxycholic acid; (6) patients with depressive disorders undergoing antidepressant treatment (selective serotonin reuptake inhibitors, serotonin–norepinephrine reuptake inhibitors, and tricyclic antidepressants) and serotonin receptor agonists. In subjects taking other medications, blood was sampled in the morning just before taking medicines.

### 2.2. Sample Size

Accepting an α risk of 0.05 and a β risk of less than 0.2 in a bilateral contrast, 24 subjects per group are needed to detect a difference ≥0.2 units. It is assumed that the common standard deviation is 0.3.

### 2.3. Liver Pathological Features

Liver samples were scored by experienced hepatopathologists using methods described elsewhere [39]. According to their liver pathological features, women with MO were subclassified into three groups: NL (n = 22), SS (micro/macrovesicular steatosis without inflammation or fibrosis, n = 21) and NASH (Brunt Grades 1–3, n = 15).

### 2.4. Biochemical Analyses

All of the subjects included underwent physical, anthropometric, and biochemical assessments. Blood samples were obtained from patients with MO and control subjects. Biochemical parameters were analyzed using a conventional automated analyzer after 12 h of fasting. Insulin resistance (IR) was estimated using the homeostasis model assessment of IR (HOMA2-IR). 

Blood extraction, which was obtained from either the group with MO or the control group, was performed by specialized nurses through a BD Vacutainer^®^ system after overnight fasting. Venous blood samples were centrifuged within 30 min on ice after collection at 3500 rpm at 4 °C for 15 min. The serum aliquots were stored at −80 °C. The serum 5-HT levels were analyzed by an enzyme-linked immunosorbent assay (ELISA) according to the manufacturer’s instructions (Ref. BA E-8900, Labor Diagnostika Nord, Nordhorn, Germany). Cytokines such as IL-1, IL-6, IL-8, TNF-α, and adiponectin were determined using multiplex sandwich immunoassays, the MILLIPLEX MAP Human Adipokine Magnetic Bead Panel 1 (HADK1MAG-61K, Millipore, Billerica, MA, USA), the MILLIPLEX MAP Human High-Sensitivity T Cell Panel (HSTCMAG28SK, Millipore, Billerica, MA, USA), and the Bio-Plex 200 instrument at the Center for Omic Sciences (Universitat Rovira i Virgili) according to the manufacturer’s instructions.

### 2.5. mRNA Levels in the Liver and Jejunum

The liver and jejunal samples collected during bariatric surgery were conserved in RNAlater (Qiagen, Hilden, Germany) at 4 °C and then processed and stored at −80 °C. The total RNA was extracted from both tissues using the RNeasy mini kit (Qiagen, Barcelona, Spain). Reverse transcription to cDNA was performed with the High Capacity RNA-to-cDNA Kit (Applied Biosystems, Madrid, Spain). Real-time quantitative PCR (RT-qPCR) was performed with the TaqMan Assay predesigned by Applied Biosystems (Foster City, CA, USA) for the detection of HTR2A, HTR2B, and HTR3 in the liver and HTR3 and HTR4 in the jejunum. The mRNA levels of each gene were calculated relative to the expression of 18S RNA for liver genes and glyceraldehyde-3-phosphate dehydrogenase (GAPDH) for genes in the jejunum, using the control group (NL) as a calibrator. All the reactions were carried out in duplicate in 96-well plates using the 7900HT Fast Real-Time PCR system (Applied Biosystems, Madrid, Spain).

### 2.6. Statistical Analysis

The data were analyzed using the SPSS/PC+ for Windows statistical package (version 23.0; SPSS, Chicago, IL, USA). The Kolmogorov–Smirnov test was used to assess the distribution of variables. Continuous variables were reported as the mean ± SD, while non-continuous variables were reported as the median and 25–75th percentile. The different comparative analyses were performed using a nonparametric Mann–Whitney U test or Kruskal–Wallis test, according to the presence of two or more groups. The strength of the association between the variables was calculated using Pearson’s method (parametric variables) and Spearman’s rho correlation test (nonparametric variables). *p* < 0.05 was considered statistically significant. GraphPad Prism 5 for Windows software (version 5.03) was used to elaborate the figures, and the statistical analysis of the relative abundance of analyzed genes was carried out the Wilcoxon test.

## 3. Results

### 3.1. Baseline Characteristics of Subjects

The baseline characteristics of the subjects given in Table 1 show the anthropometric and biochemical parameters, expressed as the mean and standard deviation or as the median and percentiles, depending on the distribution of the variables. First, we classified the subjects into two groups according to their body mass index (BMI): normal weight (NW) (BMI < 25 kg/m^2^; n = 26) and MO (BMI > 40 kg/m^2^; n = 58). Biochemical analyses showed that the women with MO had significantly higher levels of fasting glucose, insulin, homeostasis model assessment of insulin resistance (HOMA2-IR), triglycerides (TG), aspartate aminotransferase (AST), alanine aminotransferase (ALT), gamma-glutamyltransferase (GGT), and alkaline phosphatase (ALP) (*p* < 0.05) than women with NW. Moreover, the high-density lipoprotein cholesterol (HDL-C) level was significantly lower in patients with MO than in subjects with NW (*p* < 0.001). There was no difference in the rest of the lipid profile, because the patients with MO were taking lipid-lowering drugs.

Then, our cohort of women with MO was subclassified based on their hepatic histology: first as normal liver (NL, n = 22) and NAFLD (n = 36) histology; second, the patients were sub-classified into NL, SS (n = 21), and NASH (n = 15) groups.

In terms of age and anthropometric measurements (weight, BMI), there were no significant differences between the NL, SS, and NASH patients in the MO group. The laboratory parameters indicated that the glucose and alkaline phosphatase (ALP) levels were increased in SS women compared to NL and NASH women with MO. In the same way, alanine aminotransferase (ALT) was decreased in the NL group compared to SS women with MO. Finally, the glucose and GPT levels were significantly increased in women with NAFLD compared to NL.

### 3.2. Peripheral Levels of Serotonin in the Studied Cohort

**Figure 2:**
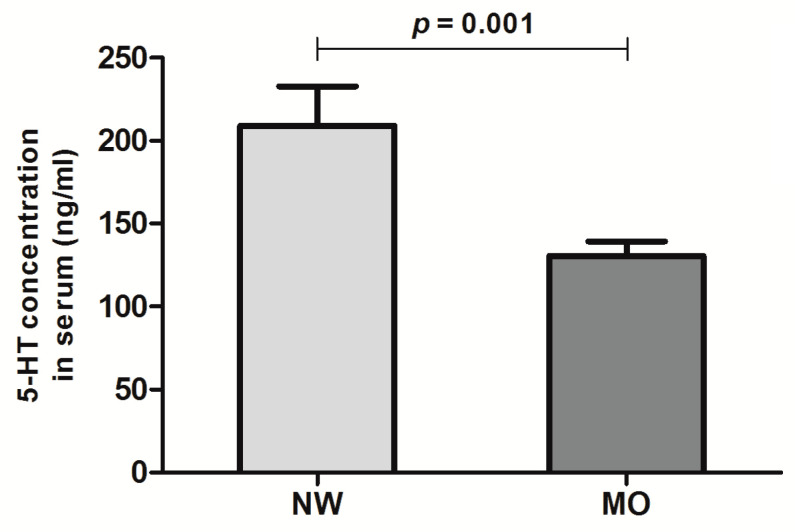
Serum levels of 5-HT in women with normal weight (NW) and women with morbid obesity (MO). *p* < 0.05 is considered statistically significant.

We analyzed the serum levels of 5-HT in both the NW controls and subjects with MO. We found that the peripheral 5-HT was reduced in women with MO compared to women with NW (*p* = 0.001, Figure 2). When we analyzed the serum levels of 5-HT according to the liver histology structure (NL vs. NAFLD), there were no significant differences (*p* = 0.700).

### 3.3. Correlations of Serum Levels of Serotonin with Metabolic Syndrome Presence

Given that the peripheral 5-HT levels have been related to obesity and T2DM, we analyzed the association between the peripheral levels of 5-HT and the presence of metabolic syndrome in our cohort. Metabolic syndrome presence was defined according to the Alberti et al. criteria described elsewhere [40]. We observed a negative correlation between them (rho = −0.334, *p* = 0.003). In this regard, the peripheral 5-HT levels correlated positively with the levels of HDL-C (rho = 0.271, *p* = 0.028) and negatively with triglycerides (rho = −0.251, *p* = 0.04). Unfortunately, we did not find any correlation between the serum levels of 5-HT and fasting glucose, systolic blood pressure (SBP), nor waist circumference (WC) (data not shown).

### 3.4. Evaluation of HTR2A, HTR2B, and HTR3 mRNA Levels in Liver and HTR3 and HTR4 in Jejunum According to Liver Histology

**Figure 3:**
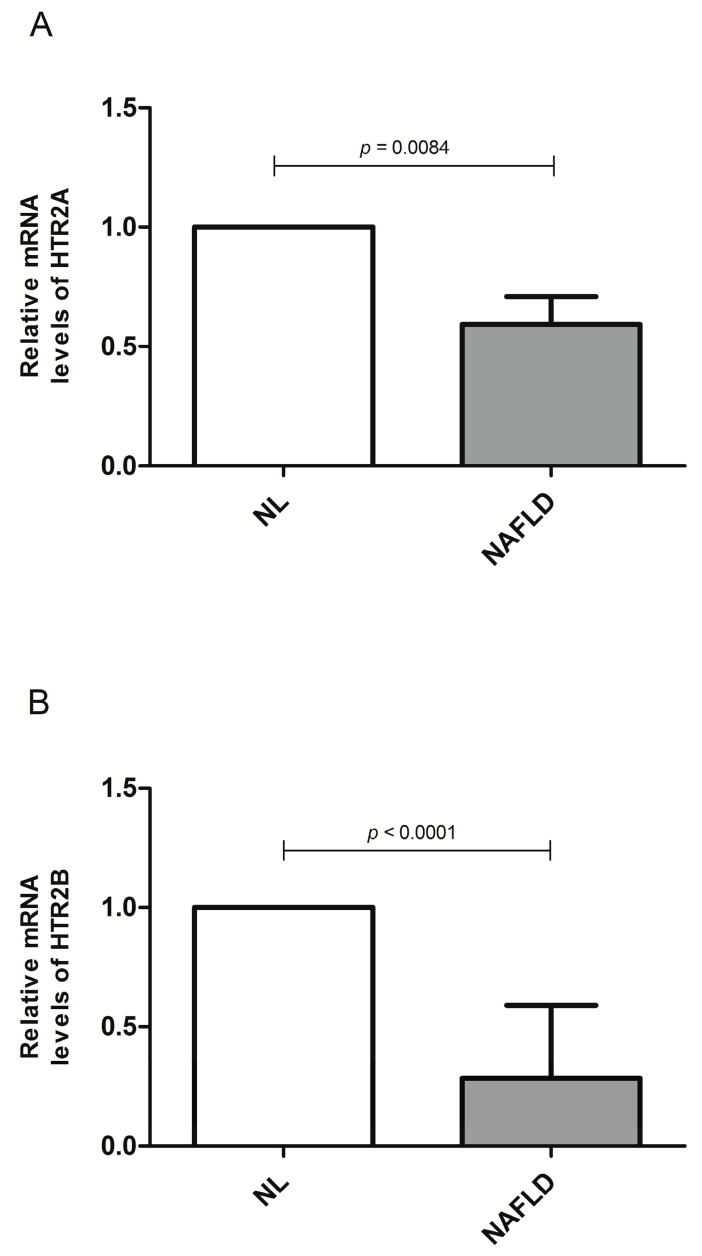
(**A**) Differential relative mRNA levels of HTR2A and (**B**) relative mRNA levels of HTR2B between women with MO with NL histology and women with MO with NAFLD. Relative mRNA abundance was expressed as the fold-change in NAFLD vs. control (2^−ΔCt NAFLD^/2^−ΔCt NL^). NL; normal liver; NAFLD, non-alcoholic fatty liver disease. *p* < 0.05 was considered statistically significant.

In the studied population of women with MO, we analyzed the relative abundance of HTR2A, HTR2B, and HTR3 mRNA in the liver. Our results showed that NAFLD women had decreased mRNA relative levels of HTR2A and HTR2B compared to the NL control group (Figure 3a,b).

**Figure 4:**
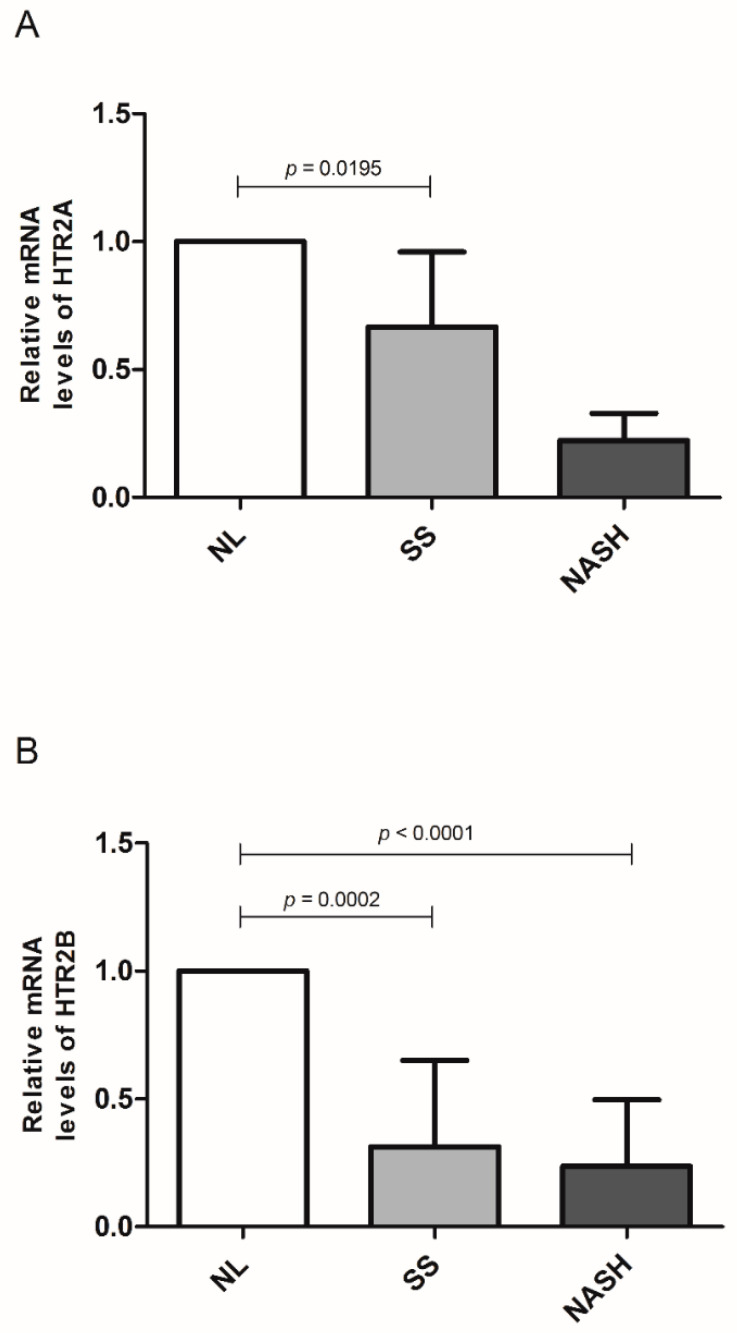
(**A**) Differential relative mRNA levels of HTR2A and (**B**) relative mRNA levels of HTR2B between women with SS or NASH compared to the NL control group. Relative mRNA abundance was expressed as the fold-change SS or NASH groups vs. the control group (2^−ΔCt SS or NASH^/2^−ΔCt NL^). NL; normal liver; NASH, non-alcoholic steatohepatitis; SS, simple steatosis. *p* < 0.05 was considered statistically significant.

Finally, we sub-classify our cohort into NL, SS, and NASH groups. We found that the HTR2A relative levels of mRNA were lower in SS compared to NL (Figure 4a). We have not found significant differences between the NL and NASH groups (*p* = 0.0625) because we had to remove some outliers in the NASH group using the Tukey correction test. The cohort obtained in the NASH group was too much small to compare with. On the other hand, the relative mRNA levels of HTR2B were significantly decreased in women with SS or NASH compared to the control group (Figure 4b).

To add new information about the role that plays intestinal receptors (HTR3 and HTR4) in the pathogenesis of NAFLD, we studied their jejunal mRNA abundance according to the liver histology. There were no significant differences in the relative mRNA levels of HTR3 and HTR4 according to the liver histology in women with MO (data not shown).

### 3.5. Correlations of Liver Expression of HTR2A, HTR2B, and HTR3 and Jejunal Expression of HTR3 and HTR4 with Serum 5-HT Levels and Metabolic Syndrome Presence

We could not find any correlation between the liver HTR2A, HTR2B, and HTR3 expression and the jejunal expression of HTR3 and HTR4 with the peripheral 5-HT levels (data not shown).

No correlation was found between the liver expression of HTR2A, HTR2B, and HTR3 and the jejunal expression of HTR3 and HTR4 with metabolic syndrome presence (data not shown).

### 3.6. Correlations of Peripheral Levels of Serotonin with Inflammatory Cytokines

As the circulating levels of some pro-inflammatory cytokines seems to be directly related to NAFLD [41,42,43,44,45], we studied the correlation between the serum levels of serotonin with the cytokine circulating levels. The serum 5-HT levels correlated negatively with the levels of interleukin (IL)-1 (rho = −0.399, *p* < 0.001) and tumor necrosis factor alpha (TNF-α) (rho = −0.287, *p* = 0.011). However, no correlation was found between the 5-HT levels and the circulating interleukins (IL-6, IL-8, C-reactive protein (CRP), and adiponectin).

## 4. Discussion

The novelty of the present study lies in the fact that we analyzed the peripheral 5-HT levels in a well-characterized cohort of women with MO and NAFLD in relation to the hepatic and jejunal mRNA abundance of HTR. We found that the peripheral 5-HT levels were lower in women with MO compared to NW subjects. Moreover, a decreased expression of hepatic HTR2B was found in women with MO and NAFLD.

First, we analyzed the serotonin peripheral levels according to the presence of obesity. We found that the 5-HT levels were decreased in women with MO. Regarding the serotonin levels in obesity, the evidence described in the literature is controversial. Some studies agree with our results and reported a negative association between the serotonin levels and weight or BMI [33,46]. According to these authors, circulating serotonin interacts with leptin in the adipose tissue and increases the feeling of satiety; therefore, it is believed that serotonin has a protective role against obesity [33]. However, other studies reported that high levels of serum 5-HT are related to obesity in animal models [35,47]. These authors describe that a HFD promotes the over-expression of TPH1, which increases the circulating serotonin levels. Moreover, in humans, it has been shown that carbohydrate-rich meals and duodenal glucose infusions enhance the 5-HT release from the gut [34,48]. This serotonin reaches tissues such as the liver, adipose tissue, or the pancreas and promotes gluconeogenesis and lipogenesis, which induce obesity [49]. The controversies arising from these articles regarding obesity and the circulating levels of 5-HT could be explained for different reasons: 1) Each study was carried out using different determination methods (HPLC, ELISA, fluorometry...) [33], which creates an important variability measurement [50]. 2) It is also possible that this variation may result from differences in diet before the measurement. Studies that reported higher levels of serotonin in subjects with obesity included a study cohort that has previously been fed a carbohydrate-rich diet or has been infused with glucose [34,48]. The same occurs in animal models in which obesity has been induced with a HFD [35,47]. Conversely, our cohort is made up of women with MO who have undergone bariatric surgery and were on a VLCD during the three months prior to surgery. It has been described that serotonin decreases with a low-calorie diet [46,51]. 3) We also have to consider that other studies, unlike ours, have been carried out with a heterogeneous cohort of men and women with a different range of age or BMI [33,34,48].

Second, we observed a negative association between the serum 5-HT levels and the presence of metabolic syndrome. Accordingly, the serum 5-HT levels correlated positively with HDL-C and negatively with triglycerides. Serotonin, this multifunctional bioamine, is synthesized in EC cells that generate all of the peripheral 5-HT. 5-HT has a local role in modulating gastrointestinal motility, but gut-derived 5-HT has also been involved in the regulation of glucose homeostasis; lipid metabolism; bone density; and diseases associated with metabolic syndrome, such as obesity and T2DM [52,53], via intestinal dysbiosis [54,55].

We also analyzed the correlation between the 5-HT levels and the circulating levels of inflammatory cytokines. In this regard, our results have shown a negative correlation between the circulating 5-HT and the levels of IL-1 and TNF-α, two important pro-inflammatory cytokines. Our results were supported by evidence from Ritze et al., who described that the circulating 5-HT levels were lower in subjects with MO compared to the NW group, while inflammatory markers were increased [46]. All these findings suggest that serotonin may have a protective role in obesity.

As intestinal dysbiosis can generate altered gut-derived metabolites that have been related to NAFLD [56] and also disrupts gut homeostasis which may affect serotonin synthesis [57], we also wanted to analyze serotonin levels according to the presence of NAFLD. There is evidence that the gut-derived serotonin reaches the liver, promoting gluconeogenesis and lipogenesis, which induce hepatic steatosis [58]. However, we were unable to find any relationship between these factors.

One of the novelties of the present work is the analysis of the expression of serotonin hepatic (HTR2A, HTR2B, and HTR3) and jejunal receptors (HTR3 and HTR4) in a cohort of women with MO and NAFLD. Our findings indicated that the hepatic HTR2A and HTR2B mRNA abundance was significantly lower in women with MO and NAFLD than the control group (NL with MO). There are several studies describing the role of serotonin receptors in hepatic steatosis, but all these studies have been assessed in animal models or cell cultures. Tsuchida and Friedman reported that the hepatic stellate cells (HSCs) involved in NAFLD progression seem to be activated by serotonin [59]. It has also been seen that inhibition of HTR2A and HTR2B reduces proliferation, increases serotonin-induced apoptosis [60] and can attenuate steatosis and fibrosis [61,62]. Recently, it was shown that the inhibition of HTR2A signaling in vivo by blocking 5-HT synthesis ameliorates hepatic steatosis [28]. Furthermore, it has also been described that HTR3 antagonist can avoid pathological processes such as lipid deposition in the liver, attenuating NAFLD progression [58]. In addition, HTR2B antagonist seems to block the serotonin-mediated activation of Notch signaling and autophagy, suggesting that HTR2B could be implicated in serotonin-mediated Notch activation, promoting cell steatosis in HepG2 cells [29]. However, Sumara et al. observed that HTR2B activation by serotonin during periods of fasting appears to regulate glucose production by promoting liver gluconeogenesis and inhibits glucose uptake [25]. Moreover, Cataldo et al. showed that pharmacological prolonged HTR2B activation reduces glucose-stimulated insulin secretion in MIN6 cells (cell line derived from a mouse insulinoma), probably due to an impaired mitochondrial activity and ATP production by mechanisms likely dependent on enhanced peroxisome proliferator-activated receptor gamma coactivator 1-alpha (PGC1α/PPARy) levels [63], improving insulin-resistance and, consequently, fatty accumulation in the liver [64]. Given the previous evidence that 5-HT-derived ROS play a key role in the pathogenesis of diet-induced steatohepatitis in murine models [65], we subclassified our cohort of women with MO into NL, SS, and NASH groups, according their liver damage. Unfortunately, in the present study we did not find any relationship between the hepatic HTR expression and NASH presence.

Moreover, the jejunal HTR expression was analyzed for the first time in women with NAFLD in the present study. In a previous work of our group, we reported that the intestine–liver axis is very important in NAFLD [8]. As stated previously, intestinal dysbiosis disrupts gut homeostasis and may affect serotonin synthesis [57]. This fact could be related to liver metabolism [52] and NAFLD development [58]. Serotonergic signaling seemed to play a prominent role in the duodenal mucosa, with high expression levels of HTR3 and HTR4 [31,66]. Ritze et al. provided the first evidence of a jejunal dysregulation of neuroendocrine markers through serotonin system in humans with severe obesity [66]. According to previous studies in mice [67] and humans with obesity [66], it has been speculated that 5-HT could promote the development of metabolic syndrome consequences, such as inflammation and fatty liver disease. Despite this, in the current study there were no significant differences in the jejunal expression levels of HTR3 and HTR4 according to the presence of NAFLD.

Some limitations should be considered. Although our cohort made it possible to establish a clear relationship between women with morbid obesity and NAFLD with deregulated hepatic HTR2B expression, these results cannot be extrapolated to overweight subjects. Additionally, because sex differences have been described in metabolic disorders and NAFLD, we have performed our study including only women in order to avoid the interference of several confounding factors such as gender. Thus, the results cannot be extrapolated to men. Moreover, our study lacks a control group of patients with MO under a standard diet to determine with certainty whether the serotonin levels in obesity are influenced by diet. Other limitations of our study are that we only examined expression profiles in jejunum samples; therefore, the findings may not be representative of the entire intestinal HTR expression. The determination of peripheral 5-HT levels probably does not reflect the platelet serotonin levels.

In summary, the present work has added some evidence regarding the role of serum serotonin and its receptor signaling in NAFLD, although some controversies remain because there are not enough reports in humans. Further studies are needed to progress in clarifying the role of serotonin and HTR in NAFLD pathogenesis.

## 5. Conclusions

Peripheral serotonin levels are decreased in women with morbid obesity under a hypocaloric diet. The negative correlation between serotonin levels and metabolic syndrome criteria suggests a protective effect on obesity. Finally, we provide the first evidence of the dysregulation of hepatic HTR2A and HTR2B mRNA abundance in women with MO and NAFLD, which could suggest a new therapeutic target.

## Figures and Tables

**Table 1:** Anthropometric and biochemical variables of the study cohort classified according to their BMI and histopathological characteristics.

**Variables**	**NW (n = 26)** **Mean ± SD**	**MO (n = 58)** **Mean ± SD**	**NL (n = 22)** **Mean ± SD**	**SS (n = 21)** **Mean ± SD**	**NASH (n = 15)** **Mean ± SD**
Age (years)	49.91 ± 9.47	45.08 ± 10.85	42.73 ± 10.01	45.45 ± 12.42	48.00 ± 9.52
WC (cm^2^)	NA	125.10 ± 11.71	124.08 ± 6.69	130.07 ± 12.39	120.42 ± 13.66
Cholesterol (mg/dl)	180.74 ± 14.27	170.41 ± 35.59	168.20 ± 36.25	171.56 ± 31.60	171.30 ± 8.62
LDL–C (mg/dL)	95.60 ± 28.18	136.34 ± 74.74	106.40 ± 36.21	99.78 ± 25.00	95.88 ± 30.27
ALP (U/L)	54.78 ± 4.90 *	67.84 ± 14.22	63.54 ± 11.54 ^§^	75.72 ± 13.76 ^#^	61.23 ± 12.76
	**Med (25th–75th)**	**Med (25th–75th)**	**Med (25th–75th)**	**Med (25th–75th)**	**Med (25th–75th)**
Weight (kg)	58.00	116.40	117.50	116.80	113.50
	(52.75–60.38) *	(108.00–130.10)	(110.25–132.00)	(110.00–130.20)	(104.00–124.00)
BMI (kg/m^2^)	21.63	43.90	43.30	44.59	44.95
	(20.07–24.04) *	(40.89–46.85)	(40.89–47.53)	(40.84–46.81)	(41.14–48.83)
Glucose (mg/dL)	80.00	92.00	85.50	102.00	99.00
	(73.00–85.00) *	(80.50–107.00)	(76.75–93.00) ^§^	(83.00–153.00)	(83.00–106.00)
Insulin (mUI/L)	5.66	9.42	9.43	9.80	6.57
	(4.94–7.89) *	(5.54–15.80)	(4.04–17.63)	(6.94–15.75)	(5.09–17.48)
HOMA2–IR	0.72	1.19	1.23	1.32	0.86
	(0.62–1.01) *	(0.71–2.27)	(0.46–2.27)	(0.93–2.28)	(0.61–2.42)
HbA1c (%)	5.40	5.60	5.40	5.60	5.60
	(5.20–5.70)	(5.30–5.98)	(5.30–5.70)	(5.30–6.10)	(5.20–6.33)
HDL–C (mg/dL)	68.00	38.50	39.00	45.00	38.00
	(59.00–81.00) *	(33.00–47.00)	(32.00–46.00)	(33.75–47.50)	(33.50–48.50)
TG (mg/dL)	62.00	123.00	105.00	128.00	128.00
	(49.00–71.00) *	(86.00–166.00)	(86.00–141.00)	(82.25–186.25)	(83.75–187.50)
AST (U/L)	17.00	23.00	20.00	27.00	30.00
	(14.00–22.00) *	(17.00–37.00)	(16.00–31.50)	(20.00–43.00)	(17.00–44.00)
ALT (U/L)	16.00	28.50	21.00	32.50	33.00
	(12.00–20.00) *	(18.00–35.00)	(15.50–30.00) ^§^	(24.25–35.75)	(16.00–30.50)
GGT (U/L)	13.00	22.00	21.00	22.00	26.00
	(11.00–17.00) *	(16.00–31.00)	(16.00–29.00)	(16.25–31.75)	(12.50–30.50)
SBP (mmHg)	120.00	118.00	122.50	121.00	115.00
	(109.00–122.00)	(107.00–133.00)	(99.25–132.75)	(110.00–140.00)	(101.50–125.25)
DBP (mmHg)	65.00	66.00	70.50	62.00	66.00
	(65.00–66.00)	(59.00–75.50)	(56.75–79.50)	(59.00–77.00)	(59.00–71.50)
RBC (10^12^/L)	4.22 (4.09–4.45)	4.26 (3.94–4.45)	4.19 (3.89–4.43)	4.20 (3.91–4.41)	4.24 (3.93–4.49)
WBC (10^9^/L)	6.00 (5.24–7.00) *	7.55 (6.17–9.12)	6.13 (5.55–9.03)	7.57 (6.38–9.37)	7.17 (5.90–9.07)
PLT (10^9^/L)	269.00	255.00	228.00	256.00	247.50
	(230.00–326.00)	(218.00–310.00)	(188.00–285.00)	(234.00–318.00)	(200.50–304.25)
HGB (g/dL)	13.00	12.10	12.10	12.05	11.95
	(12.00–13.40) *	(11.00–13.00)	(11.90–12.60)	(11.30–12.77)	(10.72–13.17)
HCT (%)	37.80	36.00	35.60	35.90	35.85
	(36.00–39.00) *	(32.90–38.50)	(34.20–38.00)	(33.42–37.92)	(32.42–38.90)

MO, morbid obesity; NW, normal weight; NL, normal liver; SS, simple steatosis; NASH, non-alcoholic steatohepatitis; NA, not available; BMI, body mass index; WC, waist circumference; HOMA2-IR, homeostatic model assessment method insulin resistance; HbA1c, glycosylated hemoglobin; HDL-C, high density lipoprotein cholesterol; LDL-C, low density lipoprotein cholesterol; TG, triglycerides; AST, aspartate aminotransferase; ALT, alanine aminotransferase; GGT, gamma-glutamyltransferase; ALP, alkaline phosphatase; SBP, systolic blood pressure, DBP, diastolic blood pressure; RBC, red blood cells; WBC, white blood cells; PLT, platelets; HGB, hemoglobin; HCT, hematocrit. Data of parametric variables are expressed as the mean ± SD. Data of non-parametric variables are expressed as median (25th and 75th percentiles). * Significant differences between the NW controls and the group with MO (*p* < 0.05). ^§^ Significant differences between the patients with NL and SS (*p* < 0.05). ^#^ Significant differences between SS and NASH (*p* < 0.05).

## Abbreviations

ALP	alkaline phosphatase
ALT	alanine aminotransferase
AST	aspartate aminotransferase
ATP	adenosine triphosphate
BMI	body mass index
CRP	C-reactive protein
DBP	diastolic blood pressure
DPP-4	dipeptidyl peptidase-4
EC	enterochromaffin cells
ELISA	enzyme-linked immunosorbent assay
GAPDH	glyceraldehyde-3-phosphate dehydrogenase
GGT	gamma-glutamyltransferase
GI	gastrointestinal
GLP-1	glucagon-like peptide-1
HbA1c	glycosylated hemoglobin
HCT	hematocrit
HDL-C	high-density lipoprotein cholesterol
HepG2	human hepatocellular carcinoma
HFD	high-fat diet
HGB	hemoglobin
HOMA2-IR	homeostasis model assessment of insulin resistance
HPLC	high-performance liquid chromatography
HSCs	hepatic stellate cells
5-HT	5-hydroxytryptamin (serotonin)
HTR2A	serotonin receptor 2A
HTR2B	serotonin receptor 2B
HTR3	serotonin receptor 3
HTR4	serotonin receptor 4
HTRx	serotonin receptor subtypes
IBS	irritable bowel syndrome
IL	interleukin
IR	insulin resistance
LDL-C	low-density lipoprotein-cholesterol
MO	morbid obesity
NAFLD	non-alcoholic fatty liver disease
NASH	non-alcoholic steatohepatitis
NL	normal liver
NW	normal weight
PGC1α/PPARy	peroxisome proliferator- activated receptor gamma coactivator 1-alpha
PLT	platelets
RBC	red blood cells
ROS	reactive oxygen species
RT-qPCR	real-time quantitative polymerase chain reaction
SBP	systolic blood pressure
SERT	serotonin transporter
SS	simple steatosis
T2DM	type 2 diabetes mellitus
TG	triglycerides
TNF-α	tumor necrosis factor alpha
TPH1	tryptophan hydroxylase 1
VLCD	very low calorie diet
WBC	white blood cells
WC	waist circumference
